# Estimation of delay to diagnosis and incidence in HIV using indirect evidence of infection dates

**DOI:** 10.1186/s12874-018-0522-x

**Published:** 2018-06-27

**Authors:** Oliver T. Stirrup, David T. Dunn

**Affiliations:** Centre for Clinical Research in Infection and Sexual Health, Institute for Global Health, University College London, Gower Street, London, WC1E 6BT UK

**Keywords:** Bayesian analysis, Diagnosis delay, HIV, Incidence estimation, Latent variables

## Abstract

**Background:**

Minimisation of the delay to diagnosis is critical to achieving optimal outcomes for HIV patients and to limiting the potential for further onward infections. However, investigation of diagnosis delay is hampered by the fact that in most newly diagnosed patients the exact timing of infection cannot be determined and so inferences must be drawn from biomarker data.

**Methods:**

We develop a Bayesian statistical model to evaluate delay-to-diagnosis distributions in HIV patients without known infection date, based on viral sequence genetic diversity and longitudinal viral load and CD4 count data. The delay to diagnosis is treated as a random variable for each patient and their biomarker data are modelled relative to the true time elapsed since infection, with this dependence used to obtain a posterior distribution for the delay to diagnosis. Data from a national seroconverter cohort with infection date known to within ± 6 months, linked to a database of viral sequences, are used to calibrate the model parameters. An exponential survival model is implemented that allows general inferences regarding diagnosis delay and pooling of information across groups of patients. If diagnoses are only observed within a given window period, then it is necessary to also model incidence as a function of time; we suggest a pragmatic approach to this problem when dealing with data from an established epidemic. The model developed is used to investigate delay-to-diagnosis distributions in men who have sex with men diagnosed with HIV in London in the period 2009–2013 with unknown date of infection.

**Results:**

Cross-validation and simulation analyses indicate that the models developed provide more accurate information regarding the timing of infection than does CD4 count-based estimation. Delay-to-diagnosis distributions were estimated in the London cohort, and substantial differences were observed according to ethnicity.

**Conclusion:**

The combination of all available biomarker data with pooled estimation of the distribution of diagnosis-delays allows for more precise prediction of the true timing of infection in individual patients, and the models developed also provide useful population-level information.

**Electronic supplementary material:**

The online version of this article (10.1186/s12874-018-0522-x) contains supplementary material, which is available to authorized users.

## Background

The majority of patients diagnosed with type-1 human immunodeficiency virus (HIV) are not identified in the primary stage of infection [1]. Some patients undergo regular testing for HIV, and so their test history can be used to determine an interval of time within which infection must have occurred; such ‘seroconverter’ cohorts have been the focus of much research on disease progression from infection [2, 3]. However, for most new diagnoses patients do not have a history of regular testing and so there can be considerable uncertainty with regards to the timing of their infection. Knowledge of the delay from infection to diagnosis is critical for public health monitoring of testing strategies and for estimation of the probable number of undiagnosed infections in a given population. There has been a renewed focus on early diagnosis of HIV as a public health priority in recent years, following the reporting of randomised trials that have definitively shown a reduction in transmission [4] and improvements in clinical outcomes [5] resulting from earlier initiation of antiretroviral therapy (ART). However, there is a statistical challenge in inferring the timing of infection using only biomarker data obtained after diagnosis.

The term ‘seroconversion’ describes the appearance of HIV antibodies in a patient’s blood, which are detected by screening tests for HIV. In a minority of patients, the timing of seroconversion can be accurately dated because they either presented with seroconversion illness or they underwent laboratory tests during the seroconversion period that definitively indicate the acute stage of infection. For ‘seroprevalent’ patients not diagnosed in acute infection (before or during seroconversion) and without a record of recent negative tests, the use of ‘recent infection testing algorithm’ (RITA) methods (based on antibody levels or affinity) can give an indication of whether they are likely to have recently contracted HIV, with cut-offs for the tests typically defined to identify infections within the previous 3–6 months [6]. However, the use of such tests is limited by imperfect performance in identifying recent infections using a fixed cut-off [7], and by the fact that they do not provide information regarding the precise timing of infections that were not ‘recent’. Furthermore, when carrying out epidemiological research using observational cohorts of patients, the availability of historic information on RITA tests may be limited.

For epidemiological studies and public health monitoring, CD4+ cell count at diagnosis is the most commonly used biomarker to assess the likely delay from infection to diagnosis [8–10]. CD4+ cells are a class of white blood cell that is gradually depleted in untreated HIV+ patients, and so relatively lower values indicate a probable greater delay in diagnosis. The CD4+ cell count is an important prognostic marker, meaning that monitoring is well integrated into the national surveillance systems for a number of countries [1, 10]. The decline in CD4+ cell count in untreated HIV infection has commonly been modelled as linear, on a square-root scale, in terms of time since seroconversion, with a ‘random intercepts and slopes’ model used to account for inter-patient differences in the value at seroconversion and rate of decline [3]. However, CD4+ cell counts show considerable variability between individuals and over time, and it has been shown that models that also contain less deterministic stochastic process elements can provide a better fit to this biomarker in treatment-naïve HIV+ patients [11, 12]; this raises questions regarding the precision with which CD4+ cell counts alone can be used to identify the probable true date of HIV infection.

It has been reported that measures of viral genetic diversity may also provide valuable information regarding the extent of the delay from infection to diagnosis of HIV. It is thought that in most patients HIV infection results from a ‘founder virus’ of a single genotype [13]. HIV is known to be a very rapidly evolving pathogen and mutations occur within an untreated individual infected with the virus, leading to an increase in viral diversity over time [14]. Classical bulk sequencing used for HIV drug resistance testing does not provide full information regarding the array of viral sequences present in an individual, but Kouyos et al. [15] found that the proportion of ambiguous nucleotide calls provides a useful proxy for viral diversity and hence also acts as an indicator for the time elapsed since infection. Similar findings have been replicated in other cohorts [16, 17].

Meixenberger et al. previously evaluated the combination of data on ambiguous nucleotide calls in *pol* sequences with RITA immunoassay results, viral load and CD4+ cell counts in identifying patients with recent HIV infection and found no benefit in combining multiple markers in comparison to the use of their RITA immunoassay alone [18]. However, in the method of analysis used, optimal fixed cut-offs for each variable were defined in order to generate dichotomous predictions as to whether the infection of each given patient was ‘recent’ or not, and it may be possible to extract more useful information from the data through statistical modelling of variables on their original continuous scales.

We aimed to develop a statistical framework that would make full use of all available clinical information in estimating the delay to diagnosis, and hence the true date, of a patient’s HIV infection. We derive parameter distributions using models for biomarker data that do not assume exact knowledge of a true date of infection even in seroconverters, and we generate full posterior distributions in evaluating the probable date of infection conditional on the clinical data in individual patients. Furthermore, we demonstrate a novel method to investigate the distribution of times from infection to diagnosis in any given subgroup of patients. This method involves estimation of the incidence of new infections as a function of time, building on models previously used to investigate the progression from transfusion-linked HIV infection to AIDS, with the potential for further public health applications.

## Methods

We first provide a general outline of the methodology that we have developed to investigate diagnosis delays in HIV. We initially fit a model to biomarker data in terms of time since infection in a ‘calibration’ dataset of seroconverters in whom we have strong information regarding the date of infection; this is done in order to characterise the ‘natural history’ of the biomarkers in untreated patients. Using this fitted model, we can make inferences regarding the timing of infection in a seroprevalent patient given their observed biomarker data and date of diagnosis. In order to do this we also need to consider whether we can make any prior assumptions regarding the likely infection date before looking at the biomarker data, and one simple approach is to assume that the date of infection is equally likely for any point in time from the legal age of sexual consent until diagnosis (termed a ‘uniform prior distribution’). However, we further develop a method that explicitly models the average diagnosis delay within a group of patients using a survival distribution. This is all done within a Bayesian framework.

The modelling approach developed is applied to data from clinical cohort studies and evaluated using simulation analyses. The established method of CD4 back-estimation of infection dates [8] is used for comparison throughout.

### Biomarker models

The model for longitudinal observations of pre-treatment CD4 counts follows the structure as described by Stirrup et al. [19]. Briefly, CD4 counts are modelled on the square-root scale, using a statistical model that includes random intercept and slope components, independent measurement error terms and a fractional Brownian motion stochastic process component. An interlinked model for pre-treatment VL measurements (on log10-scale) is used based on that proposed by Pantazis et al. [20, 21]. The proportion of ambiguous nucleotide calls at first treatment-naïve viral sequence is modelled using a zero-inflated beta model. This effectively comprises a logistic regression model for the occurrence of no ambiguous calls and a model for a beta-distributed variable amongst those cases with any ambiguous calls observed.

CD4, VL and sequence ambiguity are all modelled in terms of the ‘true time elapsed from date of infection’ in each patient. For those patients in whom this is not known exactly, this variable is formed by the sum of ‘time from diagnosis to observation’ and an unobserved latent variable representing the delay from infection to diagnosis (denoted *τ*_*i*_ for the *i*^th^ patient). For the calibration dataset *τ*_*i*_ is given a uniform prior distribution over an interval equal to the time between last negative and first positive HIV-1 tests in each patient, and for seroprevalent patients two different options for the prior are considered: a uniform prior or a prior implicit in a joint model for HIV incidence and delay to diagnosis.

We are interested in epidemiological analysis on a scale of months and years, and so do not distinguish between dates of infection and seroconversion. Further model and computational details are given in Additional file 1: Appendix A.

### Individual patient predictions with uniform priors

The biomarker model fitted to the calibration dataset is used to generate distributions for the delay to diagnosis in seroprevalent patients. We approximate the posterior distribution for all of the biomarker model parameters resulting from the calibration dataset using a multivariate normal distribution, and then use this as the prior for these model parameters in subsequent analyses for new patients.

When evaluating the delay to diagnosis in each individual new seroprevalent patient, we initially use a uniform prior distribution for this latent variable (*τ*_*i*_), defined between zero and an upper limit equal to the time elapsed between the patient’s 16^th^ birthday (or 1^st^ Jan. 1980, whichever, is later) and the date of their HIV diagnosis. The model for the observed CD4 counts, VL measurements and sequence ambiguity in each new patient is dependent on the value of *τ*_*i*_ as for the model fitted to the calibration dataset, although the range of possible values is wider. Information regarding the probable diagnosis delay is obtained by generating the posterior distribution of *τ*_*i*_ for each patient given their observed biomarker data. We employ this approach to generate predictions for one patient at a time (i.e. separate statistical models are generated and processed for each patient, although this can be run in parallel for cohorts of patients using cluster computers).

### Survival models for delay to diagnosis

In making population-level inferences, there is a problem that some patients have little biomarker data available or have biomarker values that only provide limited information regarding the timing of infection. We address this issue through the fitting of an exponential survival model for diagnosis following HIV infection. This approach enables information to be pooled across similar patients, and also allows direct investigation of patient characteristics associated with the delay to diagnosis in cases of HIV. In these analyses the approximate multivariate normal prior distribution for biomarker parameters resulting from the calibration dataset is used as previously described, but data from the entire subgroup of interest of newly observed seroprevalent patients are combined in a single statistical model.

The event time in the survival models fitted is defined as the time from HIV infection to diagnosis, once again specified as an unobserved latent variable (*τ*_*i*_) with value restricted to lie between zero and an upper limit equal to the time difference between the patient’s 16^th^ birthday (or 1^st^ Jan. 1980) and the date of their HIV diagnosis. However, the prior distribution of *τ*_*i*_ is implicit in a statistical model for HIV incidence and diagnosis. As when using a uniform prior distribution for *τ*_*i*_, for each seroprevalent patient biomarker data are modelled in terms of the true time elapsed from date of infection and this allows a posterior distribution for the delay in diagnosis to be obtained that is conditional on this information.

We can, of course, only include patients in whom HIV has been diagnosed in the analysis, and so there is no censoring of survival times. However, for a cohort of patients diagnosed in any given calendar period there is both left and right truncation of the event times. In this setting it is also necessary to model the incidence rate of new HIV infections in the population of interest. We define the start and end of the study period as *T*_*L*_ and *T*_*R*_, respectively, and denote the point in calender time of HIV infection in the *i*^th^ patient as *t*_*i*_. The left truncation results from the fact that any given patient can only be included in the cohort if *T*_*L*_<*t*_*i*_+*τ*_*i*_. The right truncation results from the fact that a patient will only be observed if *t*_*i*_+*τ*_*i*_<*T*_*R*_. This situation is directly analogous to the problem of estimating the distribution of incubation time from transfusion-acquired HIV infection to AIDS, an important issue at the start of the HIV epidemic, in which there was left truncation of observations due to a lack of recording of very early AIDS cases and right truncation due to the fact that transfusion events leading to HIV infection could only be identified retrospectively upon the development of AIDS [22]; we develop our model for the incidence rate of new HIV cases and the delay-to-diagnosis distribution based on the work of Medley et al. [23, 24] in this previous context, and we use notation also based on that employed by Kalbfleisch and Lawless [25].

Following Medley et al. [23, 24] and Kalbfleisch and Lawless [25], initiating events (i.e. HIV infections) occur according to a Poisson process for which the rate of new events is a function of time; in technical terms we define an intensity function for the process *h*(*x*;***α***),*x*>−*∞*, where *x* is a variable representing calender time and the intensity function *h*(*x*) is determined by parameter vector ***α***. We assume that the delay to diagnosis *τ* is independent of the time of infection *x*, with cumulative distribution function *F*(*τ*) and density function *f*(*τ*)=*dF*(*τ*)/*dτ*. Medley et al. [23, 24] and Kalbfleisch and Lawless [25] considered the situation at the start of an epidemic, with observation of diagnoses at any point in time up to the end of the analysis (i.e. the period (−*∞*,*T*_*R*_]) and the first non-zero incidence at a defined point in time (set to 0). However, we are interested in modelling populations in later stages of the HIV epidemic and so only consider diagnoses occurring within a defined period [*T*_*L*_,*T*_*R*_], without specifying a start time for the epidemic. We do not consider the possibility that a new HIV infection is never diagnosed (e.g. due to death before diagnosis), but believe that the proportion of such cases would be very small in the population of interest. The joint log-likelihood (*ℓ*) function, omitting dependence on model parameters, for the incidence and observation of HIV cases is then:

$$\begin{array}{*{20}l} \ell &= \sum_{i=1}^{n} \left\{\log \left(h\left(x_{i} \right) \right) + \log \left(f\left(\tau_{i} \right) \right) \right\} - A, \\ \text{where,}\ A&= \int_{-\infty}^{T_{R}} h\left(x \right) \left\{ F\left(T_{R}-x \right) - F\left(T_{L}-x \right) \right\} dx, \end{array} $$

$$\begin{array}{*{20}l} &= \int_{-\infty}^{T_{L}} h\left(x \right) \left\{ F\left(T_{R}-x \right) - F\left(T_{L}-x \right) \right\} dx \\&\quad+ \int_{T_{L}}^{T_{R}} h \left(x \right) F\left(T_{R}-x \right) dx. \end{array} $$

This matches the form of the expression used previously [23–25], but the integral denoted *A* is adjusted to reflect the truncated observation window and the lack of assumptions regarding the start of the epidemic.

It is noted by Kalbfleisch and Lawless [25] that the absolute incidence of new infections can be eliminated from the joint likelihood function by conditioning on the total number of cases observed. However, it is still necessary to model the relative incidence as a function of calendar time unless constant incidence can be assumed at all points up until the end of the study period. The assumption of constant incidence might be justified for a completely stable endemic disease, but this condition is not common in the epidemiology of infectious diseases.

We are primarily interested in fitting a model for the delay-to-diagnosis distribution, but in doing so we are therefore required to model the incidence of new infections prior to and during the calender period under investigation. Ideally the function for the incidence of new HIV cases, *h*(*x*), would be chosen so as to provide a plausible representation of the entire epidemic. However, when attempting to fit models to data from patients diagnosed decades after the start of the epidemic, this is not a practical objective. Instead, we propose a pragmatic approach in which the incidence (*h*(*x*)) is assumed to be either exponentially increasing or decreasing prior to the calender period of interest (i.e. for *x*<*T*_*L*_), and to be either constant or in a separately defined state of exponential change during the period itself (i.e. for *T*_*L*_<*x*<*T*_*R*_). We therefore define the incidence rate function as either:

$$\begin{array}{*{20}l} \text{1:} &h\left(x \right) = e^{\left(c + \delta_{1} \left(x \right) b\left(x-T_{L} \right) \right)}, \text{ or} \\ \text{2:} &h\left(x \right) = e^{\left(c + \delta_{1} \left(x \right) b\left(x-T_{L} \right)+ \delta_{2} \left(x \right) d\left(x-T_{L} \right) \right)}, \end{array} $$

where the function *δ*_1_(*x*)=1 if *x*<*T*_*L*_ and 0 otherwise, *δ*_2_(*x*)=1 if *x*>*T*_*L*_ and 0 otherwise, and *c*, *b* and *d* are model parameters: exp(*c*) is the incidence rate at *T*_*L*_, *b* determines the rate of decay (*b*<0) or growth (*b*>0) of incidence prior to this and *d* (in ‘Option 2’) determines the change in incidence after *T*_*L*_. For an exponential model for the delay-to-diagnosis distribution with rate parameter *λ*, for *b*+*λ*>0 the integral required for the log-likelihood function can be solved analytically in each case (results in Additional file 1: Appendix B).

The functions that we have suggested for *h*(*x*) clearly cannot provide a full description of the HIV epidemic. However, we propose that allowing for an increasing or decreasing trend in HIV incidence directly prior to the period of interest will appropriately adjust for truncation of diagnosis dates as long as the function *h*(*x*) provides an adequate description across the probable range of infection dates of the patients included in the analysis. The first option presented assumes constant incidence of new HIV infections during the observation period, which may be appropriate for short analysis windows, whilst the second option also allows a change in incidence following the start of the observation period. Further computational details are given in Additional file 1: Appendix B.

### Datasets and software used

We present analyses that make use of viral sequences of the protease and reverse transcriptase regions of the *pol* gene collected by the UK HIV Drug Resistance Database [26] that can be linked to pseudo-anonymised clinical records of patients enrolled in the UK Collaborative HIV Cohort (UK CHIC) [27] and UK Register of Seroconverters (UKR) cohort [2]. The statistical methodology was developed using a ‘calibration’ dataset comprising 1299 seroconverter patients from the UKR cohort who can be linked to a treatment-naïve partial *pol* sequence. All patients included from the UKR cohort have an interval between last negative and first positive HIV tests of less than 1 year, and some patients were identified during primary infection, meaning that their date of infection can be treated as fixed and known. Injecting drug users were excluded from the analysis.

The methodology developed was then applied to a seroprevalent cohort of men who have sex with men (MSM) diagnosed with HIV in London over a 5-year period spanning 2009–2013 and enrolled in the UK CHIC study. We only included men with an age of at least 18 years at time of diagnosis with a treatment-naïve partial *pol* sequence stored in the UK HIV Drug Resistance Database. We also excluded any men enrolled in the UKR study. This led to a sample size of 3521 patients. Pre-treatment CD4 counts and VL measurements were included in the analysis, but were not considered as part of the inclusion criteria.

We employ a fully Bayesian approach, implemented in the Stan probabilistic programming language [28]. We carried out all Bayesian modelling using a Linux cluster computer; although fitting individual models using a modern desktop computer would be feasible. The authors acknowledge the use of the UCL Legion High Performance Computing Facility (Legion@UCL), and associated support services, in the completion of this work. Maximum likelihood estimation of random effects models was performed using the *lme4* package for R; these were used in the CD4 back-estimation of infection dates performed for comparison.

### Cross-validation analysis

We performed a cross-validation analysis using the calibration dataset of seroconverters in order to evaluate the performance of our methodology. We split the calibration dataset into five test groups of nearly equal size (i.e. 259 or 260 patients per group) and refitted the biomarker model five times, excluding one of the test groups on each occasion. The resulting biomarker model fit was then used to generate predictions regarding the timing of HIV seroconversion in the excluded group for each iteration as if they were seroprevalent patients, i.e. disregarding any knowledge regarding the precise timing of infection or history of negative HIV tests. We initially used a uniform prior distribution for time of infection (from the patient’s 16^th^ birthday to date of HIV diagnosis) when generating predictions, and also fitted exponential survival models for the delay to diagnosis pooled across the test group in each case (without the need to account for truncation of observations). Maximum likelihood estimation of a standard ‘random intercepts and slopes’ model for CD4 counts was also carried out alongside Bayesian fitting of the biomarker model at each iteration, and predictions were generated in the test group using simple CD4 back-estimation as described by Rice et al. [8].

### Simulation analyses

To further investigate the properties of the methodology developed, we carried out several simulation analyses. Firstly, we generated data for 2000 hypothetical patients with unknown date of HIV infection without considering the truncation of observation times. For this purpose we set distributional parameters equal to the posterior mean values obtained when our model was fitted to the calibration dataset without the inclusion of lab-specific random effects and, to further simplify matters, data were only generated for white MSM with subtype-B HIV acquired at the age of 32. The delay from infection to diagnosis was set to follow an exponential distribution with rate parameter of 0.5 (on the scale of years). Nucleotide ambiguity proportions were simulated at the time of diagnosis, and CD4 counts and VL measurements were generated at time of infection, after 1.5 months, 3 months and subsequently at 6-month intervals from 6 months to 3 years. If a negative CD4 count was generated, then this and all subsequent simulated clinic visits were censored; this meant that a few simulated patients were excluded completely and so a new patient was generated to replace them. The limit of detection for VL was set to 50 copies/mL in the simulations. We initially used a uniform prior distribution for time of infection (from the patient’s 16^th^ birthday to date of HIV diagnosis) when generating predictions, and also fitted an exponential survival model for the delay to diagnosis pooled across simulated patients. Simple CD4 back-estimation based on a fitted ‘random intercepts and slopes’ model was used for comparison [8].

Two additional simulation analyses were carried out with time-varying incidence and truncation of observation times. Patients were generated with characteristics, delays to diagnosis and scheduled set of viral sequence, CD4 and VL observations as described for the simulation of observations without truncation. However, incidence was varied over a measure of calender time, and patients were only selected for analysis whose simulated date of diagnosis fell within a specified analysis window. Models were fitted with estimation of both the rate of diagnosis parameter (*λ*) and incidence rate over calendar time, allowing the latter to vary before and during the analysis window. Firstly a simulated cohort was generated with increasing incidence from zero for 10 years prior to the analysis window, and a constant incidence rate of 200/year during the analysis window of 5 years’ duration. Secondly a simulated cohort was generated with constant incidence rate of 300/year for 10 years, followed by a decrease to 150/year over the 5 years prior to the analysis window and a further decrease to 100/year over the 5 years of the analysis window itself.

## Results

### Biomarker model in calibration dataset

When the biomarker model was fitted to the calibration dataset it was found that most patient and viral characteristics did not show any clear association with the proportion of ambiguous nucleotide calls on viral sequencing, with the 90% credibility intervals (CrI) for these parameters including zero, and so most of these parameters were dropped from the model to simplify it. There was one exception in that male patients infected via heterosexual sex are more likely to have zero ambiguous nucleotide calls (95% CrI for parameter on logit-scale: 0.06–1.36, in model without lab effects), and so this was retained in the model reported and used for subsequent analysis.

**Fig. 1:**
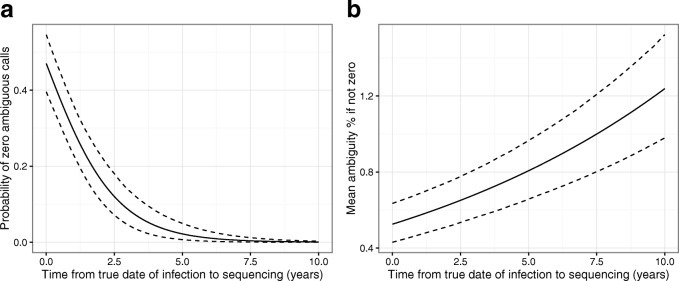
Viral sequence ambiguity relative to time since infection. Plot of **a** probability of zero ambiguous calls and **b** mean ambiguity percentage *if not zero* as a function of time from true date of infection to sequencing (in years). The functions plotted are defined for men who have sex with men or heterosexual women with viral sequencing at a ‘typical’ lab (with lab specific variations set to zero). Plots are displayed of the expected function value (black line) and 95% credibility interval (dashed line) over the joint posterior distribution of relevant model parameters as resulting from the calibration dataset

Some substantial inter-lab differences were observed in the probability of observing a viral sequence with zero ambiguity calls, and so lab-specific random effects (as described in Additional file 1: Appendix A) are retained in all analyses. We describe the relationship between time elapsed from HIV infection to viral sequencing and the proportion of ambiguous nucleotide calls for a ‘typical’ lab, with the three lab-specific random effect terms set to zero. Immediately following HIV infection there is an estimated probability of zero ambiguous nucleotide calls on viral sequencing of just below 0.5, but this probability drops to close to zero for sequences obtained beyond around 5 years from the date of infection (Fig. 1a). There is a corresponding increase in the mean percentage of ambiguous nucleotide calls, amongst those patients in whom any are observed, from around 0.5% immediately following infection to around 1.2% 10 years after infection (Fig. 1b). Further details and summaries of the posterior distributions for all model parameters, including those for CD4 counts and VL, are provided in Additional file 1: Appendix C.

### Investigation of delay to diagnosis in seroprevalent cohort

The delay-to-diagnosis distribution was investigated in the cohort of 3521 seroprevalent MSM. We first generated predictions for the delay to diagnosis of each individual patient using a uniform prior (in combination with the biomarker model). The overall mean of the posterior expectation in each patient was 4.12 years, and divided by ethnicity it was 3.99 years for white (*n*=2577), 4.58 years for black (*n*=239) and 4.46 years for mixed/other/unknown (*n*=705) patients. For those patients in whom at least one CD4 count was available, the overall mean diagnosis delay estimated by CD4 back-estimation was 2.87 years (*n*=3414), and divided by ethnicity it was 2.59 years for white (*n*=2501), 3.97 years for black (*n*=233) and 3.56 years for mixed/other/unknown (*n*=680) patients.

**Fig. 2:**
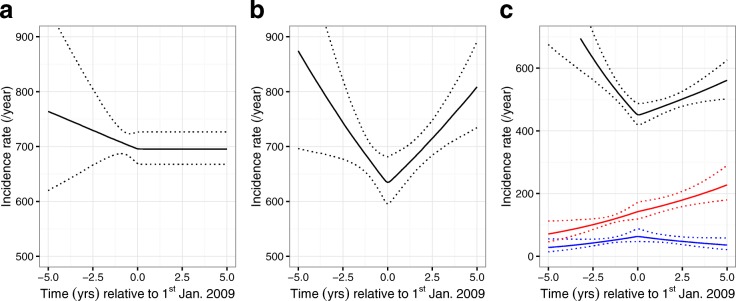
Plots of estimated incidence rates (/year) of new HIV infections. Results from models fitted to the cohort of 3521 seroprevalent men who have sex with men. In **a** the incidence of new HIV infections is assumed to be constant in the window period, whereas in **b** and **c** it is allowed to vary. In **a** and **b**, a single delay-to-diagnosis distribution is fitted across all patients, whereas in **c** it is split according to white (black line), black (blue line) and other (red line) ethnic classifications. 95% credibility intervals are shown as dotted lines

The use of ‘incidence and delay-to-diagnosis’ models fitted to the entire dataset of seroprevalent patients revealed a similar pattern of differences between subgroups defined by ethnicity as found using uniform priors or CD4 back-estimation, but the estimates of average delay were consistently lower (Table 1). When a constant incidence of HIV was assumed during the window period and a single delay-to-diagnosis distribution was fitted across all ethnic groups (Fig. 2a), the posterior mean estimate of average time (1/*λ*) from infection to diagnosis was 1.82 years (95% CrI 1.64–2.04 years), and allowing the incidence of HIV to change during the window period led to only a small change in the estimate to 1.77 years (95% CrI 1.59–1.96 years) even though a change in incidence was found during this period (Fig. 2b). The second model was further extended to allow differences in the delay-to-diagnosis distribution according to ethnicity, and patients of black (2.91, 95% CrI 1.92–4.76 years) or other (2.68, 95% CrI 2.04–3.45 years) ethnicity were found to have substantially higher average time-to-diagnosis than white patients (1.57, 95% CrI 1.41–1.75 years). The ethnic classifications of patients also showed differences in incidence trends over time (Fig. 2c). Further computational details and examples of predictions for the date of infection in individual patients are presented in Additional file 1: Appendix D.

**Table 1:** Estimates of mean diagnosis delay (years) in the cohort of 3521 seroprevalent men who have sex with men diagnosed in London in the period 2009–2013 using the models developed in this paper and by CD4 back-estimation

		By patient ethnicity		
Model	Overall	White	Black	Mixed/other/unknown
CD4 back-estimation^a^	2.87	2.59	3.97	3.56
Full biomarker model with uniform priors^b^	4.12	3.99	4.58	4.46
Full biomarker model with exponential survival model for diagnosis				
Constant incidence during analysis window, no division by ethnicity^c^	1.82 (1.64–2.04)	—	—	—
Changing incidence during analysis window, no division by ethnicity^c^	1.77 (1.59–1.96)	—	—	—
Changing incidence during analysis window, with division by ethnicity^c^	—	1.57 (1.41–1.75)	2.91 (1.92–4.76)	2.68 (2.04–3.45)

^a^Mean of point estimates

^b^Mean of posterior expectation in each individual patient

^c^Estimated as part of model with associated 95% CrI

### Results of cross-validation analysis

**Fig. 3:**
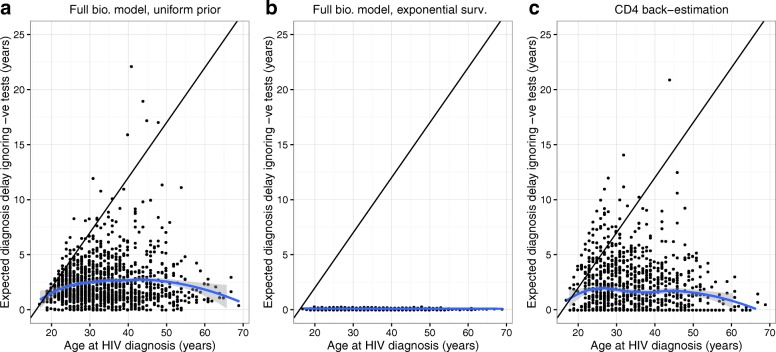
Plot of predictions of delay to diagnosis resulting from the cross-validation analysis. Results are shown in relation to patient age at diagnosis, and are presented using our methodology (full biomarker (bio.) model; for which ∙ is the posterior expectation) with **a** uniform priors or **b** a pooled exponential survival (surv.) model in each test group and **c** using standard CD4 back-estimation. The diagonal black line shows the ‘expected’ diagnosis delay for a uniform prior distribution from the age of 16 to the date of diagnosis in each patient. LOESS regression curves are also shown (blue line) with 95% CI (shaded grey). The maximum true diagnosis delay in these patients is 1 year

The cross-validation analysis made use of the calibration dataset of seroconverters and so the maximum possible true diagnosis delay in these patients is 1 year. However, when our methodology was used with a uniform prior distribution for the delay to diagnosis of each patient the mean estimated diagnosis delay was 2.45 years (taking the mean of posterior expectations) and the interquartile range was 1.16–3.26 years (Fig. 3a). This was worse than the performance of simple CD4 back-estimation for which the mean estimated delay to diagnosis was 1.71 years with an interquartile range of 0.00–2.90 years (Fig. 3c). Plots are presented of the estimated diagnosis delay against patient age at diagnosis because the time period from start of sexual activity to date of diagnosis represents the maximum possible delay from infection to diagnosis, and so there is the potential for greater delays amongst patients who are older at diagnosis.

When an exponential survival model was used for the delay to diagnosis, individual patient estimates for the diagnosis delay were appropriately corrected into the range 0–1 years (Fig. 3b). This performance was mediated by high values for the posterior distribution of the rate of diagnosis parameter (*λ*), with posterior mean ranging from 9.2–12.6 across the test groups (corresponding to a mean delay to diagnosis of 4–6 weeks). The mean estimated diagnosis delay was 0.01 years and the interquartile range was 0.07–0.12 years; average delays around this level are not likely to be observed in patients with unknown date of infection, but these results demonstrate how the methodology developed can successfully pool information across a group of patients.

### Results of simulation analyses

#### Without truncation of observation times

**Fig. 4:**
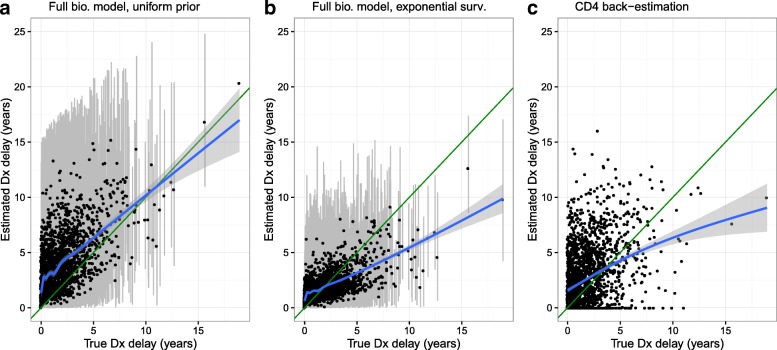
Predictions of delay to diagnosis (Dx) against true delay from the simulation analysis without truncation. Results are presented using our methodology (for which ∙ is the posterior expectation with 95% credibility interval in grey) with **a** uniform priors or **b** a pooled exponential survival (surv.) model and **c** using standard CD4 back-estimation. The diagonal green line shows the line of equality for perfect predictions in each patient. LOESS regression curves are also shown (blue line) with 95% CI (shaded grey)

The results of the simulation analysis for 2000 patients without truncation of observation times are summarised in Fig. 4 and Table 2. The use of our methodology with a correctly defined exponential survival model showed the best accuracy for individual patient predictions for the delay to diagnosis: taking the posterior mean for each individual as a point estimate gave a mean absolute error of 1.04 years and a mean squared error of 2.15, with values of 2.22 years and 8.39 for the use of our methodology with uniform priors and 2.12 years and 8.40 for CD4 back-estimation.

**Table 2:** Summary of accuracy of delay-to-diagnosis predictions for the simulation analysis of 2000 patients without truncation of observation times. Our methodology was applied using either uniform priors or an exponential survival model for diagnosis delays, and CD4 back-estimation was used for comparison

	Full biomarker model, uniform prior	Full biomarker model, exponential survival model	CD4 back-estimation
Absolute error			
Mean (years)	2.22	1.04	2.12
Lower quartile (years)	0.79	0.37	0.67
Median(years)	1.73	0.75	1.54
Upper quartile(years)	3.24	1.40	2.98
Mean squared error (years^2^)	8.39	2.15	8.40
Bias (mean error) (years)	1.97	–0.02	0.68
Coverage of 95% CrI^a^	89.5	94.1	NA

^a^coverage of 95% credibility intervals for posterior distribution of the diagnosis delay in individual patients (relative to known true value in simulation)

For the model with exponential survival distribution individual patient predictions show ‘shrinkage’ towards the population average of 2 years, explaining the consistent overestimation for smaller true delays and underestimation for larger true delays, but the mean bias was very close to zero (-0.02 years). However, consistent overestimation was observed when uniform priors were used (reflecting a larger prior expected value for the delay), with a mean bias of 1.97 years. Furthermore, the posterior 95% CrIs for the delay to diagnosis in each patient showed correct coverage when the exponential model was used (94.1 %) but not when uniform priors were used (89.5 %). Whilst ad hoc procedures have been proposed, simple CD4 back-estimation does not incorporate a coherent method for generating confidence or credibility intervals, but the mean bias was 0.68 years reflecting an overestimation of the average diagnosis delay. The exponential survival model recovered an appropriate estimate and CrI for the rate of HIV diagnosis following infection (posterior expectation: $\hat {\lambda }=$ 0.52, 95% CrI 0.48–0.57; true value=0.5).

This simulation analysis shows that our method for estimating the delay-to-diagnosis distribution across a group of patients can lead to smaller prediction errors on a per patient basis and more accurate group-level inferences than the use of CD4 back-estimation. When our methodology was used with uniform priors for the delay-to-diagnosis distribution in each patient the performance was poor both at the individual-patient and group level, despite the use of all available pre-treatment biomarker information within the model; this shows that the use of uniform priors in the analysis of diagnosis delays can lead to very inaccurate inferences. Results have been described for a single simulated cohort for simplicity of presentation, but this simulation analysis was repeated 100 times to confirm the performance of the exponential survival model (Additional file 1: Appendix E).

#### With truncation of observation times

**Fig. 5:**
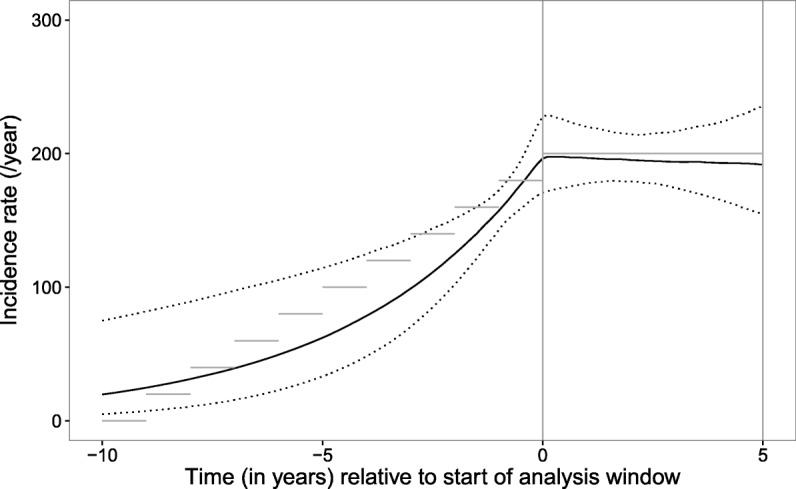
Plot of estimated incidence rate (/year) of new infections for first simulation with analysis window. The estimated incidence rate (/year) of new HIV infections (black line) is plotted with 95% credibility interval (dotted line) for simulation with increasing incidence prior to and constant incidence during the analysis window. Horizontal grey lines show the true incidence rate used to generate the data, and vertical grey lines show the limits of the analysis window

For the first simulated cohort with a truncated observation window, generated with increasing and then constant incidence, there was a total of 877 patients diagnosed during the analysis period. The incidence of new infections during the window period was estimated correctly, and the estimated trend prior to the analysis window also reflected that used to generate the data (Fig. 5). The 95% CrI for the posterior distribution for the rate of diagnosis parameter included the true value (0.42–0.64, posterior expectation =0.53; true value =0.5). When a delay-to-diagnosis model was fitted without accounting for any potential change in incidence, a posterior expectation of 0.59 (95% CrI 0.52–0.67) was obtained for this parameter. Code to generate an equivalent dataset and refit the ‘incidence and delay-to-diagnosis’ model is provided online (Additional files 2, 3, 4, and 5).

**Fig. 6:**
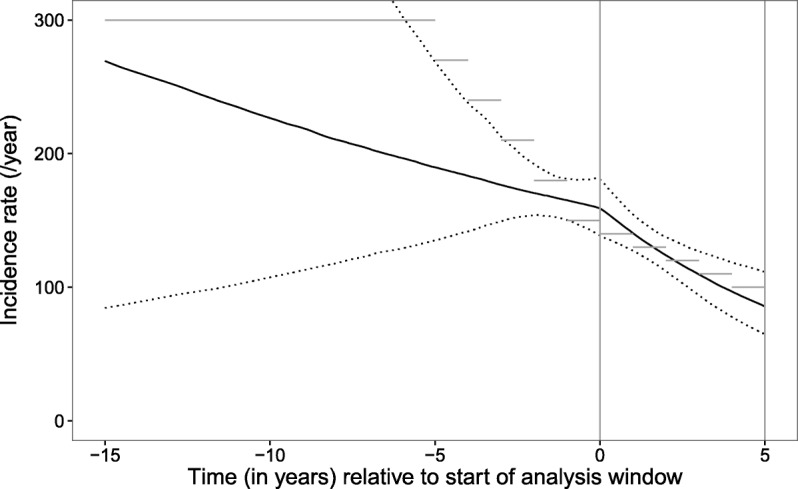
Plot of estimated incidence rate (/year) of new infections for second simulation with analysis window. The estimated incidence rate (/year) of new HIV infections (black line) is plotted with 95% credibility interval (dotted line) for simulation with decreasing incidence prior to and during the analysis window. Horizontal grey lines show the true incidence rate used to generate the data, and vertical grey lines show the limits of the analysis window

For the second simulated cohort with a truncated observation window, generated with decreasing incidence, there was a total of 721 patients diagnosed during the analysis period. The incidence of new infections during the window period was estimated correctly (Fig. 6). The incidence prior to the window period was not captured perfectly due to the constraints of the model used, but the trend estimated by the posterior mean of the model parameters did reflect that used to generate the data (with the 95% CrI indicating considerable uncertainty). The 95% CrI for the posterior distribution for the rate of diagnosis parameter included the true value (0.39–0.55, posterior expectation =0.47; true value =0.5). When a delay-to-diagnosis model was fitted without accounting for any potential change in incidence, a posterior expectation of 0.44 (95% CrI 0.39–0.48) was obtained for this parameter. These simulations demonstrate that our methodology can correctly identify trends in incidence, assuming that the model is appropriate to the data, and that it can be used to estimate the average diagnosis delay with adjustment for changes in incidence.

## Discussion

In this paper we have developed novel statistical methodology to derive probability distributions for the date of HIV infection in individual patients and to investigate the characteristics of delay-to-diagnosis distributions within a population of interest. The use of a fully Bayesian framework for statistical modelling allows the combination of multiple sources of available information and also means that uncertainty in parameter estimates can be incorporated in all stages of the analysis, without the need for bootstrapping to generate credible or confidence intervals. We have included viral nucleotide ambiguity, CD4 counts and VL measurements in the models developed, but the framework could also be readily extended to incorporate other biomarkers where available. The information that can be gained through our methodology is of direct use for public health monitoring and planning, and it may also provide a useful contribution to research into HIV transmission networks and dynamics.

In demonstrating our methods we have investigated how diagnosis delays vary with patient ethnicity among MSM in London, finding substantially greater delays to diagnosis in non-white individuals. This finding is consistent with those reported based on a crude definition of late diagnosis of CD4 count <350 within 3 months of diagnosis [29], and a similar pattern of differences was observed when we used CD4 back-estimation for comparison in our analysis. However, the average diagnosis delay for all groups was found to be lower when it was estimated using a survival model pooled across patients. Explicit estimation of the diagnosis delay distribution in subgroups of interest could be very useful for public health monitoring and in the planning of interventions such as targeted outreach testing. We should note that we have analysed a selected cohort with inclusion conditional on enrolment into the UK CHIC study and availability of a treatment-naïve viral sequence, and so the findings that we have observed cannot be used for any specific public health conclusions without further research.

There are some conceptual similarities between our approach and that developed by Sommen et al. [30] using immunological markers for recent HIV infection. Sommen et al. [30] fitted longitudinal models for HIV antigen biomarkers by maximum likelihood estimation with integration over the known possible interval of infection times in a cohort of seroconverters, and the parameter estimates obtained were then used to derive posterior distributions for the true time of infection in seroprevalent patients given their observed biomarker data using an exponential prior distribution for the delay to diagnosis; however, the parameter determining the shape of the exponential prior distribution was treated as fixed and known. Romero-Severson et al. [31] further developed the approach of Sommen et al. [30], and used a prior distribution fitted to point estimates of delay to diagnosis based on BED-enzyme immunoassay results. We have taken this approach a step further and have estimated the characteristics of the delay distribution using data from seroprevalent patients, without using intermediate point estimates, through the use of a fully Bayesian modelling framework. We could not directly apply the models developed by Sommen et al. [30] and Romero-Severson et al. [31] to our cohort, as we did not have immunoassay data available for the patients.

Both Sommen et al. [30] and Romero-Severson et al. [31] derived posterior distributions for the date of HIV infection in individual patients as a step towards generating incidence estimates within a population of interest. In the present work we have shown that modelling of incidence is required in order to appropriately estimate the delay-to-diagnosis distribution across a subgroup of patients, unless constant incidence can be assumed. The estimates of incidence resulting from our study relate to a highly selected group of patients and so are not of direct interest with regards to public health planning. However, it would be possible to apply our methodology to an unselected cohort of patients, even if some individuals have very little or no biomarker data available, in order to estimate the total incidence in a population of interest.

Several established methodological approaches to the estimation of HIV incidence from surveillance data have been developed from a Bayesian multi-state model proposed by Sweeting et al. [32], with Birrell et al. [33] and van Sighem et al. [9] providing examples of the application of such models to national cohorts. In these analyses, the rate of diagnosis following HIV infection is estimated, but data are modelled in terms of discrete time points and discrete disease stages are defined in terms of CD4 count. The models that we have developed are defined on the original continuous time scale, allowing more detailed analysis of the delay from infection to diagnosis in individual patients or across a population. Our models may allow changes in incidence to be quantified over a smaller time period, given the use of all available biomarker data in continuous form, but this requires further investigation.

There has been much interest recently in the use of phylogenetic analysis to investigate HIV transmission networks and dynamics, which requires the direction of infection in transmission pairs to be identified. For example, Ratmann et al. [34] conducted an analysis of Dutch MSM and estimated that in over 70% of infections in their selected cohort, transmission had occurred from an undiagnosed man, indicating a need for more targeted testing for HIV. Ratmann et al. used CD4 back-estimation to derive infection times in assessing direction of transmission, which could be further refined using our methodology; given the clinical and viral sequence data of a transmission pair, it would be possible to explicitly derive the probabilities for which patient was the first to be infected, and of onward transmission having occurred within a defined period of time following the initial infection. Another potential application for our methods would be to estimate the probability of infection having been acquired in another country among patients in migrant populations, as averaging over patient-specific probabilities might give different results to the use of point estimates in individual patients as has previously been performed [8].

A limitation of the present study is that RITA/antigen biomarkers were not included in the analysis. This was because of only very limited available data in the calibration dataset. However, as we have noted, it would be straightforward to incorporate such data into the framework developed. RITA/antigen biomarkers alone are generally only used to provide a dichotomous classification into recent or non-recent infection, but in combination with other clinical and genetic data it would be possible to further refine the interval within which infection is likely to have occurred in any given seroprevalent patient.

Another limitation of this study is that we only considered classical bulk Sanger sequencing of a limited segment of the viral genome, and did not include any data from ‘next generation sequencing’ (NGS) methods. This was due to the fact that historical data were used for the analysis, although it is worth noting that, in the UK, NGS has still only been introduced for clinical use in HIV in a few centres. NGS techniques can provide complete sequencing of the viral genome and also enable more in-depth assessment of viral diversity than classical Sanger sequencing, allowing phylogenetic techniques to be employed to reconstruct viral evolution from one or more founder viruses, which in turn can be used to predict the date of infection [35]. These techniques would, however, require greater computational resources for the processing of sequence data for each patient, and so measures of ambiguous nucleotide proportions may still be useful for carrying out population-level analyses.

## Conclusions

The modelling strategy developed in this paper builds on prior work but includes a novel combination of features within a single coherent framework. We have developed this approach with the aim of making full use of all available relevant data in assessing timing of infection in seroprevalent HIV patients, whilst appropriately incorporating and quantifying uncertainty in both model parameters and true dates of HIV infection at each stage of the analysis. Cross-validation and simulation analyses indicate that the models developed provide more accurate information regarding the timing of infection than does CD4 count-based estimation, and they also provide useful population-level information. The focus of the present paper is on investigation of delays to diagnosis, but we plan to further develop the application of our framework for incidence estimation.

## Additional files

Additional file 1 Supplementary Appendices. Contains further details of model specifications, computational notes, parameter summaries in the calibration dataset and examples of predictions in individual patients. (PDF 206 kb)

Additional file 2 R script (viewable as plain text) to read in parameter estimates for the calibration dataset and to then simulate a cohort of patients with increasing incidence of HIV and a limited observation window for diagnoses. The same R script fits an ‘incidence and delay-to-diagnosis’ model to the generated data using a Stan model template file provided. (R 17 kb)

Additional file 3 Comma-separated value file containing posterior mean values for parameters of model fitted to the calibration dataset (without accounting for inter-lab variation in nucleotide ambiguity proportions). (CSV 4 kb)

Additional file 4 Comma-separated value file containing posterior covariance matrix for parameters of model fitted to the calibration dataset (without accounting for inter-lab variation in nucleotide ambiguity proportions). (CSV 52 kb)

Additional file 5 Stan model template file (which is annotated and can be viewed as plain text) to fit the ‘incidence and delay-to-diagnosis’ model with change in incidence prior to and during observation window. (STAN 17 kb)

## Abbreviations

AIDS: Acquired immune deficiency syndrome
ART: Antiretroviral therapy
CrI: Credibility interval
HIV: Human immunodeficiency virus
MSM: Men who have sex with men
NGS: Next generation sequencing
RITA: Recent infection testing algorithm
UCL: University College London
UK: United Kingdom
UK CHIC: UK Collaborative HIV Cohort
UKR: UK Register of Seroconverters
VL: Viral load
