# Supplementary material for: Estimation of delay to diagnosis and incidence in HIV using indirect evidence of infection dates
Source: BMC Med Res Methodol. 2018 Jun 27;18:65. doi: 10.1186/s12874-018-0522-x (PMC6020319; doi:10.1186/s12874-018-0522-x)
Supplement: Supplementary file 1 — Supplementary Appendices. Contains further details of model specifications, computational notes, parameter summaries in the calibration dataset and examples of predictions in individual patients. (PDF 206 kb) [file 12874_2018_522_MOESM1_ESM.pdf]

Supplementary Appendices to: Estimation of delay to diagnosis and incidence in HIV using indirect evidence of infection dates

Oliver T Stirrup and David T Dunn
Centre for Clinical Research in Infection and Sexual Health,
Institute for Global Health,
University College London, London, UK

A Calibration model and prior details

A.1 Model definitions

Pre-treatment viral load (VL) is analysed on the $\log_{10}$ scale, and we make use of the non-linear model for the mean in terms of time from seroconversion as reported by Pantazis *et al.*[1]:

$$g_{VL}(t_{VL}) = \beta_{0VL} + \beta_{1VL} t_{VL} + \beta_{2VL} \exp\left(-\beta_{3VL} t_{VL}\right), \tag{1}$$

where t_{VL} is the time of VL observation from date of seroconversion and β_{0VL}–β_{3VL} are parameters. We considered the addition of further parameters related to the following patient characteristics and their interaction with time (i.e. effectively adjusting β_{0VL} and β_{1VL}): sex, mode of infection, ethnicity, age at infection (quadratic function centred at 32 years) and viral subtype.

The patient-specific random intercept and slope of VL are modelled as following a joint multivariate normal distribution with the random-intercept and -slope terms of the pre-treatment CD4 part of the model, and there is also an examination-specific independent normal error term for VL:

$$\begin{aligned}
\mathbf{v}_i|_{\tau_i} &= \mathbf{g}_{VL}(\mathbf{t}_{VL:i}) + \mathbf{1}\mathrm{b}_{VL_0:i} + \mathbf{t}_{VL:i}\mathrm{b}_{VL_1:i} + \mathbf{e}_{VL:i} \\
\mathbf{y}_i|_{\tau_i} &= \mathbf{X}_i\boldsymbol{\beta} + \mathbf{Z}_i\mathbf{b}_i + \mathbf{W}_i + \mathbf{e}_i \\
\begin{pmatrix}\mathbf{b}_{VL:i} \\ \mathbf{b}_i\end{pmatrix} &\sim MVN\left(\mathbf{0}, \begin{pmatrix}\boldsymbol{\Psi}_{\boldsymbol{VL}} & \boldsymbol{\Psi}_{\boldsymbol{VL:CD4}} \\ \boldsymbol{\Psi}_{\boldsymbol{CD4:VL}} & \boldsymbol{\Psi}\end{pmatrix}\right) \\
\mathbf{e}_{VL:i} &\sim MVN(\mathbf{0}, \sigma^2_{VL}\mathbf{I}_{n_{VL:i}}) \\
\mathbf{W}_i|_{\gamma_i,\tau_i} &\sim MVN(\mathbf{0}, \frac{1}{\gamma_i}\boldsymbol{\Sigma}_i) \\
\mathbf{e}_i &\sim MVN(\mathbf{0}, \sigma^2\mathbf{I}_{n_i}) \\
\gamma_i &\sim \text{Gamma}\left(\frac{\nu}{2}, \frac{\nu}{2}\right) \\
\tau_i &\sim \text{Uniform}(0, c_i).
\end{aligned}$$

Here, for the viral load part of the model, $\mathbf{v}_i$ is the vector of $n_{VL:i}$ pre-treatment VL observations for the ith patient at times $\mathbf{t}_{VL:i}$, $\mathbf{g}_{VL}$ is a vectorised version of the function in (1), $\mathbf{1}$ is a vector of '1's of length $n_{VL:i}$, $\mathrm{b}_{VL_0:i}$ and $\mathrm{b}_{VL_1:i}$ are the subject-specific random intercept and slope terms for VL with covariance matrix $\boldsymbol{\Psi}_{\boldsymbol{VL}}$ and $\mathbf{e}_{VL:i}$ is a vector of examination-specific residuals for VL with variance σ^2_{VL}.

CD4 counts are measured on the square-root scale, with $\mathbf{y}_i$ representing the vector of n_i pre-treatment observations for the ith individual at times $\mathbf{t}_i$. $\mathbf{X}_i$ represents the design matrix for the 'fixed effects' parameters $\boldsymbol{\beta}$, $\mathbf{Z}_i$ represents the subset of the columns of the design matrix associated with the 'random effects' for each individual $\mathbf{b}_i$ and $\mathbf{e}_i$ is the vector of residual errors for each pre-treatment measurement occasion. All models considered include 'random intercept and slopes' components for the CD4 model, i.e. with a vector of '1's and $\mathbf{t}_i$ included in both $\mathbf{X}_i$ and $\mathbf{Z}_i$. Models were also considered in which $\mathbf{X}_i$ included additional columns corresponding to the following patient and viral characteristics and their interaction with time since seroconversion: sex, mode of

infection, ethnicity, age at infection (quadratic function centred at 32 years) and viral subtype (with additional associated parameters in $\boldsymbol{\beta}$).

$\mathbf{W}_i$ denotes the vector of values for a fractional Brownian motion stochastic process at times $\mathbf{t}_i$, with $\frac{1}{\gamma_i}\boldsymbol{\Sigma}_i$ defined as the covariance matrix for the process of the ith individual, dependent on the parameters κ and H and with variance conditional on a gamma-distributed variable γ_i; allowing between-patient differences in variability over time, determined by the 'degrees of freedom' parameter ν, as previously described[2;3].

The random effects terms for the VL and CD4 parts of the models are correlated, with covariance between them defined by the $\boldsymbol{\Psi_{VL:CD4}}$ sub-matrix. The 'observation' time vectors for both viral load and CD4 counts, $\mathbf{t}_{VL:i}$ and $\mathbf{t}_i$, are defined as 'time from true date of seroconversion' and are conditioned on the delay from seroconversion to diagnosis in each patient 'τ_i'. In patients in the 'seroconverter' cohort in whom the exact date of infection is not known, τ_i is assumed to follow a uniform distribution with lower limit zero and upper limit equal to the time interval 'c_i' between the dates of last negative and first positive HIV test in the ith patient.

A further complication is that the VL measurements are truncated at lower and upper limits of detection, with these limits depending on the equipment used at each examination and ranging from 1–500 copies/mL for the lower limit and 50 000–10^8 copies/mL for the upper limit. Following Thiébaut *et al.*[4;5], we account for this issue by making use of the fact that the likelihood contribution for such an observation below a lower limit of detection, conditional on the subject-specific random effects, is independent of other observations and can be expressed using the cumulative normal distribution function (Φ)[6] and the lower limit of detection in that case (lim^L_{ij}):

$$L(v_{ij}|\mathbf{b}_{VL:i},\tau_i) = \Phi\left(\left(lim^L_{ij} - \left(g_{VL}\left(t_{VL:ij}\right) + \mathrm{b}_{VL_0:i} + t_{VL:ij}\mathrm{b}_{VL_1:i}\right)\right)/\sigma_{VL}\right),$$

while the likelihood contribution for observations above the upper limit of detection can be expressed using the upper limit (lim^U_{ij}) in that case:

$$L(v_{ij}|\mathbf{b}_{VL:i},\tau_i) = 1 - \Phi\left(\left(lim^U_{ij} - \left(g_{VL}\left(t_{VL:ij}\right) + \mathrm{b}_{VL_0:i} + t_{VL:ij}\mathrm{b}_{VL_1:i}\right)\right)/\sigma_{VL}\right).$$

Here, the subscripts denote the jth VL measurement in the ith individual.

Given the values of $\mathbf{b}_{VL:i}$, γ_i and τ_i, the likelihood function for the CD4 part of the model can be expressed in closed form; this follows the strategy detailed in the Appendix of Stirrup *et al.* (under review) using standard expressions for a conditional multivariate normal distribution. Hence, the latent variables corresponding to the random effects ($\mathbf{b}_i$) and realisations of the stochastic process ($\mathbf{W}_i$) for CD4 are not declared as parameters in the Stan model template, with the marginal log-likelihood (conditional on other latent variables) added to the log-probability for the model.

The probability density function for the zero-inflated beta distribution for the proportion of ambiguous nucleotide calls at first treatment-naïve viral sequencing (a_i) can be written as follows:

$$f_A\left(a_i|\alpha',\beta',\theta'\right) = \begin{cases} \theta' & \text{if } a_i = 0 \\ (1-\theta')\frac{1}{B(\alpha',\beta')}a_i^{\alpha'-1}(1-a_i)^{\beta'-1} & \text{if } 0 < a_i < 1, \end{cases}$$

where, $B(\cdot,\cdot)$ is the beta function. The Bernoulli distribution parameter is restricted to the range $0 < \theta' < 1$, and so is modelled using a logistic link function, whilst the beta distribution parameters are restricted to $0 < \alpha'$ and $0 < \beta'$ and so are both modelled using a log link function. The linear predictor in each case includes an intercept term and a parameter associated with the time elapsed from seroconversion to blood sampling (conditional on the delay-to-diagnosis variable in that patient), and parameters were also considered for the set of patient characteristics included in the viral load and CD4 models. Different laboratories can show consistent differences in the proportion of ambiguous nucleotide calls returned in viral sequences, and in order to adjust for this we also consider lab-specific random effects for α', β' and θ', modelled as following a multivariate normal distribution. We note that this additional level of nested latent variables would make accurate maximum likelihood estimation of the defined model very difficult, but this does not have a substantial effect on computational requirements for Bayesian model fitting.

A.2 Prior distributions for parameters

As the analysis is carried out in a fully Bayesian framework, we are required to specify prior distributions for each of the model parameters. As we feel that the calibration dataset contains enough information to inform parameter values with minimal additional input, we opt to follow a strategy of using weakly informative prior distributions[7]. The rationale for this choice is to add to computational stability whilst allowing the data under investigation to dominate the posterior distribution for each parameter obtained through model fitting.

Briefly, standard deviation parameters of CD4 and VL patient-specific random effects were given an exponential prior with rate 0.1, as were the standard deviation parameters for lab-specific random effects. The correlation matrices for both the CD4-VL and the lab-specific random effects were given an LKJ(2) prior[7;8]. The log-scale parameter for the fractional Brownian motion process and log-variance parameter for measurement error of CD4 counts were given a normal(0,5) prior, as was the logit-H parameter for fractional Brownian motion. The log-standard deviation parameter for VL measurement error was also given a normal(0,5) prior. The mean intercept and slope parameters for CD4 counts, the log of the degrees of freedom parameter (ν) for the fractional Brownian motion process and the population average parameters for $\log_{10}$(VL) were given normal distributions with mean value taken from Stirrup *et al.* (under review) and variance 5. All parameters related to the model for nucleotide ambiguity proportion were given a normal(0,5) prior, as were all 'effect' parameters linked to patient characteristics for the CD4 count, VL or sequence ambiguity aspects of the model.

A.3 Computational notes

The combined bivariate model is similar to that used for pre-treatment data in a further analysis by Stirrup *et al.*[9], but this former work only included a single random effects term for the VL model and did not allow for between-patient differences in variability over time for the CD4 model; these restrictions were necessary because of

the computational pressures that follow from the application of maximum likelihood estimation to a complex model without a closed-form log-likelihood function. In the current analysis we employ a fully Bayesian approach, implemented in the Stan probabilistic programming language[10], which places fewer limitations on model complexity. In defining a model in Stan, the user is required to write out a template for the marginal log-probability given the data in terms of a set of parameters. We follow the strategy of Stirrup *et al.* (under review) in expressing the marginal log-probability for the model with integration over the CD4-related latent variables conditional on the values of the VL-related random variables in order to improve computational performance.

A.4 Computational approach for generating predictions in new patients

One general approach for generating predictions would be to re-run Bayesian model fitting in the calibration dataset in combination with any new patient or group of patients; with analysis then performed using the posterior distribution of the delay to diagnosis in the patient(s) of interest. However, carrying out this procedure for individual patients, or small groups of patients, would be computationally inefficient (effectively requiring that the model be refitted to the calibration dataset for every run), whilst such an analysis may not be computationally feasible for a large group of patients. The approach of using a multivariate normal approximation to parameters in the biomarker model as fitted to the calibration dataset avoids refitting of the model in the calibration dataset on each occasion, and also has the advantage that inferences regarding the delay to diagnosis in a new patient could be obtained without access to the original calibration dataset.

We have carried out all analyses using the Stan software because it allows the development of complex models for which it would be very difficult to code an efficient and robust model-specific sampling program. The Stan software requires prior distributions to be specified for the parameters of any new model, and it is not possible to feed in a pre-existing sample vector. In a fully Bayesian framework, information regarding parameters can be updated by any newly available data; there is the potential for the analysis dataset to provide further useful information regarding model parameters even after the calibration stage, but this would not be possible using a fixed sample vector of model parameters. We also note that the use of the Stan software means that the model files created to run our analyses could be edited to incorporate different biomarkers or to allow application to a completely different disease area.

B Computational details for diagnosis and incidence model

In order to fit the specified model in the Stan software, in combination with the model for post-diagnosis biomarker data, we simply add the log-likelihood function:

$$\ell = \sum_{i=1}^{n} \left\{\log(h(x_i)) + \log\left(f(\tau_i)\right)\right\} - A$$

to the log-probability conditional on the current values of the parameters and the delay to diagnosis in each patient. If we use $\boldsymbol{y}$ to denote all biomarker data, $\boldsymbol{\theta}$ to denote the full vector of parameters in the calibration model and $\boldsymbol{\tau}$ to denote the full vector of delay-to-diagnosis times, then the full log-probability for the model with 'Option 2' for $h(x)$ can be expressed as:

$$\begin{aligned}\log(P) = \sum_{i=1}^{n} \{ & \log\left(f_y\left(\boldsymbol{y}_i|\boldsymbol{\theta},\gamma_i,\boldsymbol{b}_{VL:i},\tau_i\right)\right) + \log\left(f_\gamma\left(\gamma_i|\boldsymbol{\theta}\right)\right) + \log\left(f_{b:VL}\left(\boldsymbol{b}_{VL:i}|\boldsymbol{\theta}\right)\right) \\ & + \log\left(h\left(x_i|\tau_i,b^*,c,d\right)\right) + \log\left(f\left(\tau_i|\lambda\right)\right)\} \\ & + \log\left(p\left(\boldsymbol{\theta}\right)\right) + \log\left(q\left(b^*,c,d,\lambda\right)\right) - A.\end{aligned}$$

As we are fitting these models to seroprevalent patients, $p(\boldsymbol{\theta})$ represents the density function for the multivariate normal approximation to the posterior distribution of model parameters obtained from the calibration dataset (including lab-specific variation in the sequence ambiguity model), and $q(b^*,c,d,\lambda)$ is the density function for the prior distribution for the 'new' model parameters relating to incidence and delay to diagnosis. As it would be difficult to set priors for the incidence parameters without reference to the data, we use uniform priors for b^*, c and d, and a weakly informative normal distribution with SD 3 is used for the prior of $\log(\lambda)$. If we wish to fit the delay-to-diagnosis model for different subgroups, then we must define a separate set of (b^*,c,d,λ) parameters for each subgroup.

One issue in implementing this analysis in the Stan software is that it we are required to define a function for the marginal log-probability of the model as a whole that is differentiable in terms of the entire set of declared model parameters (including here the τ_i, on which the x_i are conditioned). This causes a problem in defining $\delta_1(x)$ and $\delta_2(x)$, which are discontinuous functions of τ_i (even though the likelihood surface is a continuous function of τ_i). We instead use close approximations to '$\delta_1(x)(x-T_L)$' and '$\delta_2(x)(x-T_L)$' that are differentiable in terms of τ_i in defining our model, using T_i to denote the observed time of HIV diagnosis:

$$\begin{aligned}\delta_1(x_i)(x-T_L) &\approx x_i - T_L - \frac{1}{20}\log\left(1+e^{20(x_i-T_L)}\right), \\ \delta_2(x_i)(x-T_L) &\approx \frac{1}{20}\log\left(1+e^{20(x_i-T_L)}\right), \text{ where } x_i = T_i - \tau_i.\end{aligned}$$

This creates a continuous and differentiable 'hinge' function[11] for the log of the incidence rate of new HIV infections, with the 'hinge' centred at T_L.

B.1 Under constant incidence

Absolute incidence of new infections can be eliminated from the joint likelihood function by conditioning on the total number of cases observed. However, it is still neces-

sary to model the relative incidence as a function of calendar time unless constant incidence ($h(x) = c_{inc}$) can be assumed at all points in the period $(-\infty, T_R]$; in this case the expression can be reduced to:

$$\begin{aligned}
\ell_c &= \sum_{i=1}^{n} \left\{\log(c_{inc}) + \log\left(f(\tau_i)\right)\right\} + \log(n!) - n\log(A), \\
&= \sum_{i=1}^{n} \left\{\log\left(f(\tau_i)\right)\right\} + \log(n!) - n\log(T_R - T_L),
\end{aligned}$$

as A is equal to $c_{inc}(T_R - T_L)$ for any survival distribution for the delay to diagnosis.

B.2 Integrals for the exponential diagnosis delay model

For an exponential model for the delay-to-diagnosis distribution with rate parameter λ, for $b + \lambda > 0$ the integral required for the log-likelihood function can be solved analytically for the two models of changing incidence proposed:

$$\begin{aligned}
1\colon A &= \frac{e^c - e^{c-\lambda(T_R-T_L)}}{\lambda + b} + e^c \left(\frac{e^{-\lambda(T_R-T_L)} - 1}{\lambda} + (T_R - T_L) \right), \\
2\colon A &= \frac{e^c - e^{c-\lambda(T_R-T_L)}}{\lambda + b} + e^c \frac{\left(d\left(e^{-\lambda(T_R-T_L)} - 1\right) + \lambda\left(e^{d(T_R-T_L)} - 1\right)\right)}{d(d+\lambda)}.
\end{aligned}$$

The '$b + \lambda > 0$' condition indicates that the proportional rate of decrease in incidence prior to T_L cannot be greater than the rate at which infections are diagnosed, in such a scenario the number of expected diagnoses in the calender period would be infinite under the model as defined, and so it is necessary to constrain the b parameter to impose this condition; as such, we fit the model in terms of a new parameter $b^* \in (0, \infty)$, with $b = b^* - \lambda$.

C Biomarker model summary for calibration dataset

The model described was fit to data in the calibration dataset using four chains with a total of 1250 iterations and warm-up of 500 iterations for each, giving 3000 samples from the posterior distribution of the parameter set. Convergence of the chains to a stable posterior distribution was checked by visual inspection of trace plots and evaluation of the Gelman–Rubin potential scale reduction statistic [12] using 'split chains' [10]. The four sample chains were run in parallel, and the required 'wall-clock' time was 28 hours and 20 minutes.

The posterior expectations of parameters describing the relationships between CD4 counts and VL measurements and time elapsed since HIV infection were similar to the maximum likelihood estimates obtained by Stirrup *et al.* [9], including the random effects and measurement error variance parameters (Table C1). A number of patient characteristics showed a substantial association with CD4 counts (parameters defined on square root scale): male heterosexuals showed a lower average intercept (95 % CrI, –3.12 to –0.10), black ethnicity was associated with a lower intercept (–3.23 to –0.66) but also a lower rate of decline with time (0.11 to 0.90 higher slope), the 'other' ethnicity category was also associated with a lower rate of decline (0.05 to 0.72) as was the presence of subtype A HIV (0.15 to 1.25). Patient age at infection was not associated with CD4 count intercept, but age above 32 years was associated with progressively steeper slopes of CD4 decline with time following infection (Figure C1). A number of patient characteristics also showed a substantial association with VL measurements (parameters defined on $\log_{10}$-scale): female heterosexuals had a lower VL intercept (–0.68 to –0.13) as did patients of black (–0.70 to –0.20) or 'other' (–0.46 to –0.05) ethnicity. Patient age at infection above 32 years was associated with higher VL measurement intercept, but no relationship of age at infection with VL slope was detected (Figure C2). Given the number of substantial associations observed for CD4 counts and VL measurements, parameters for all patient characteristics were retained in the models used for further analysis.

Table C1: Posterior parameter summaries of model fitted to calibration dataset

	Mean	P2.5.	P97.5.	n_eff	Rhat	Description
logit_theta[1]	-0.120	-0.424	0.184	1,698.400	1	Amb mod: int
logit_theta[2]	-0.759	-0.977	-0.562	3,000	1	Amb mod: tpar
log_alpha[1]	0.028	-0.103	0.190	1,586.300	1	Amb mod: int
log_alpha[2]	0.083	0.052	0.114	3,000	1	Amb mod: tpar
log_beta[1]	5.276	5.033	5.547	1,338.100	1	Amb mod: int
log_beta[2]	-0.003	-0.043	0.036	3,000	1	Amb mod: tpar
male_MSW_theta	0.601	-0.077	1.290	3,000	1	Amb mod: MSW para
log_kappa	1.815	1.637	1.963	179.500	1.020	CD4 mod: fBM var
logit_H	-1.829	-1.991	-1.656	273.600	1.020	CD4 mod: fBM para
log_vdf	1.440	1.247	1.630	161.200	1.030	CD4 mod: fBM df para
Omega[2,1]	-0.389	-0.488	-0.277	3,000	1	CD4-VL RE cor matrix
Omega[3,1]	-0.481	-0.537	-0.421	3,000	1	CD4-VL RE cor matrix
Omega[3,2]	0.013	-0.093	0.118	3,000	1	CD4-VL RE cor matrix
Omega[4,1]	0.322	0.225	0.410	1,161.800	1	CD4-VL RE cor matrix
Omega[4,2]	-0.705	-0.782	-0.614	1,716.500	1	CD4-VL RE cor matrix
Omega[4,3]	-0.495	-0.558	-0.424	1,019.300	1.010	CD4-VL RE cor matrix
sigmaU[1]	4.009	3.802	4.230	3,000	1	CD4 mod: int RE SD
sigmaU[2]	0.899	0.793	1.007	3,000	1	CD4 mod: slope RE SD
sigmaU[3]	0.836	0.797	0.876	3,000	1	VL mod: int RE SD
sigmaU[4]	0.239	0.219	0.261	679.600	1	VL mod: slope RE SD
log_sigma2_cd4	0.238	-0.053	0.488	211.800	1.020	CD4 mod: ME var
int_cd4	23.465	23.141	23.791	3,000	1	CD4 mod: intercept
slope_cd4	-1.266	-1.390	-1.144	1,134.200	1	CD4 mod: slope
male_MSW_int_cd4	-1.614	-3.120	-0.103	3,000	1	CD4 mod: int para
fem_MSW_int_cd4	0.797	-0.602	2.202	3,000	1	CD4 mod: int para
male_MSW_slope_cd4	-0.088	-0.655	0.493	2,205.300	1	CD4 mod: slope para
fem_MSW_slope_cd4	-0.183	-0.684	0.291	1,304.400	1	CD4 mod: slope para
age_int_cd4_1	0.002	-0.037	0.038	3,000	1	CD4 mod: int para
age_int_cd4_2	-0.515	-2.835	1.724	3,000	1	CD4 mod: int para
age_slope_cd4_1	-0.014	-0.026	-0.002	1,232.600	1	CD4 mod: slope para
age_slope_cd4_2	-0.319	-1.136	0.507	1,817.900	1	CD4 mod: slope para
black_int_cd4	-1.959	-3.228	-0.661	3,000	1	CD4 mod: int para
black_slope_cd4	0.497	0.108	0.900	1,395	1	CD4 mod: slope para
other_ethn_int_cd4	-0.335	-1.337	0.712	3,000	1	CD4 mod: int para
other_ethn_slope_cd4	0.375	0.049	0.718	972.700	1	CD4 mod: slope para
crf_subt_int_cd4	0.412	-0.454	1.277	3,000	1	CD4 mod: int para
crf_subt_slope_cd4	0.152	-0.185	0.496	1,804.700	1	CD4 mod: slope para
A_subt_int_cd4	-0.059	-1.446	1.311	3,000	1	CD4 mod: int para
A_subt_slope_cd4	0.703	0.152	1.251	2,060.100	1	CD4 mod: slope para
C_subt_int_cd4	0.223	-1.337	1.891	3,000	1	CD4 mod: int para
C_subt_slope_cd4	-0.117	-0.674	0.458	1,798.200	1	CD4 mod: slope para
other_subt_int_cd4	0.307	-1.514	2.051	3,000	1	CD4 mod: int para
other_subt_slope_cd4	0.597	-0.011	1.236	3,000	1.010	CD4 mod: slope para
log_sigma_VL	-0.748	-0.764	-0.732	3,000	1	VL mod: ME SD
beta_VL[1]	4.404	4.335	4.476	408.900	1.010	VL mod: int
beta_VL[2]	0.072	0.043	0.100	324.100	1.010	VL mod: slope
beta_VL[3]	1.825	1.731	1.925	3,000	1	VL mod: peak acute
beta_VL[4]	2.613	2.504	2.720	3,000	1	VL mod: log decl para
male_MSW_int_VL	-0.013	-0.314	0.278	490	1	VL mod: int para
fem_MSW_int_VL	-0.409	-0.684	-0.135	529.900	1.010	VL mod: int para
age_int_VL_1	0.005	-0.002	0.012	455.500	1	VL mod: int para
age_int_VL_2	0.133	-0.317	0.616	533	1	VL mod: int para
black_int_VL	-0.441	-0.699	-0.196	325.100	1.010	VL mod: int para
other_ethn_int_VL	-0.247	-0.464	-0.046	357.600	1.010	VL mod: int para
crf_subt_int_VL	0.026	-0.150	0.207	388.800	1.010	VL mod: int para
A_subt_int_VL	0.036	-0.226	0.311	643.900	1.010	VL mod: int para
C_subt_int_VL	0.099	-0.218	0.424	598.300	1	VL mod: int para
other_subt_int_VL	-0.225	-0.573	0.139	918.100	1	VL mod: int para
male_MSW_slope_VL	0.015	-0.110	0.140	1,180.900	1	VL mod: slope para
fem_MSW_slope_VL	0.037	-0.072	0.144	717	1	VL mod: slope para
age_slope_VL_1	0.001	-0.002	0.003	489.700	1.010	VL mod: slope para
age_slope_VL_2	-0.094	-0.278	0.089	619.600	1	VL mod: slope para
black_slope_VL	-0.028	-0.124	0.067	726.400	1	VL mod: slope para
other_ethn_slope_VL	-0.016	-0.096	0.065	463.200	1	VL mod: slope para
crf_subt_slope_VL	0.025	-0.049	0.101	428.100	1.010	VL mod: slope para
A_subt_slope_VL	-0.069	-0.188	0.057	1,209.400	1.010	VL mod: slope para
C_subt_slope_VL	0.020	-0.109	0.146	980.600	1	VL mod: slope para
other_subt_slope_VL	-0.001	-0.144	0.141	1,034.500	1.010	VL mod: slope para
labOmega[1,2]	-0.053	-0.686	0.635	1,179.400	1	lab RE cor matrix
labOmega[1,3]	-0.079	-0.679	0.568	1,487	1	lab RE cor matrix
labOmega[2,3]	0.358	-0.454	0.883	568	1.010	lab RE cor matrix
lab_sigma[1]	0.451	0.200	0.824	1,402.600	1	lab RE theta SD
lab_sigma[2]	0.140	0.012	0.313	711.900	1	lab RE alpha SD
lab_sigma[3]	0.322	0.077	0.603	627.100	1	lab RE beta SD

cor, correlation; decl, decline; df, degrees of freedom; int, intercept; fBM, fractional Brownian motion; ME, measurement error; MSW, men who have sex with women (heterosexual transmission); n_eff, effective sample size; para, parameter; P2.5, 2.5th percentile of posterior distribution; P97.5, 97.5th percentile of posterior distribution; RE, random effect; Rhat, Gelman–Rubin potential scale reduction statistic; tpar, interaction parameter with time; var, variance; VL, viral load.

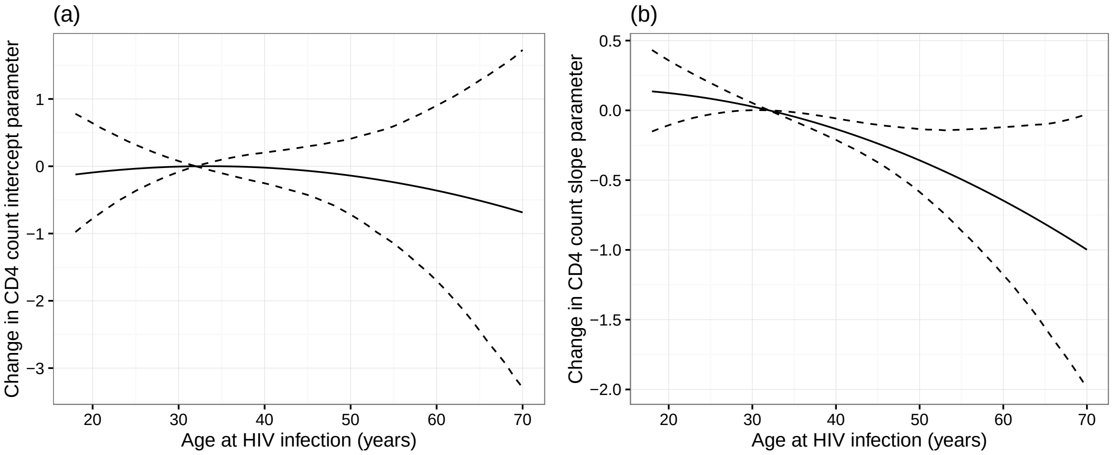

Figure C1: Plot of adjustment of CD4 count (a) intercept (value at seroconversion) and (b) slope (with time elapsed since seroconversion, in years) according to patient age at seroconversion (in years). Effects have been modelled as a quadratic function of patient age centred at 32 years, and the model for CD4 counts is defined on a square-root scale. Plots are displayed of the expected function value (black line) and 95 % credibility interval (dashed line) over the joint posterior distribution of relevant model parameters.

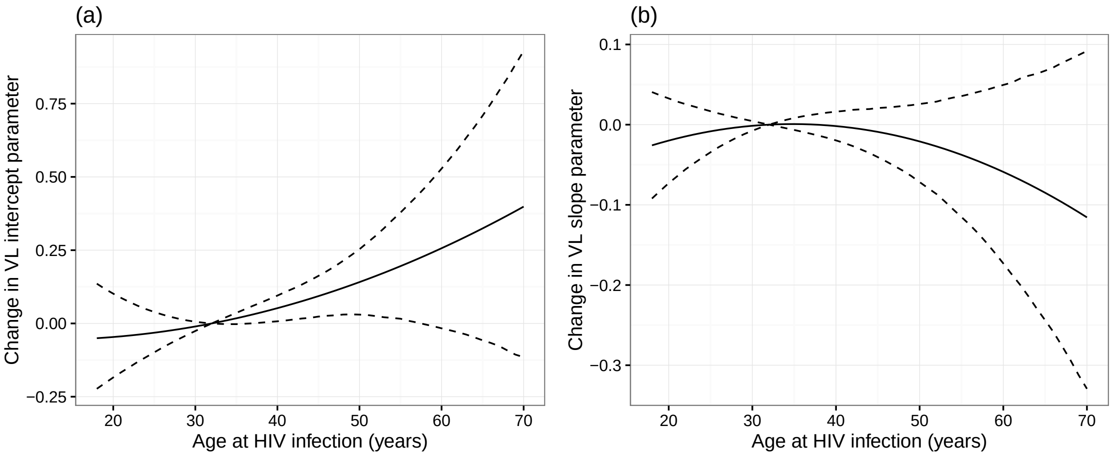

Figure C2: Plot of adjustment of viral load (VL) measurement (a) intercept (value at seroconversion) and (b) slope (with time elapsed since seroconversion, in years) according to patient age at seroconversion (in years). Effects have been modelled as a quadratic function of patient age centred at 32 years, and the model for VL measurement is defined on a $\log_{10}$-scale. Plots are displayed of the expected function value (black line) and 95 % credibility interval (dashed line) over the joint posterior distribution of relevant model parameters.

D Further details for seroprevalent cohort

As for the calibration dataset, the ‘incidence and delay-to-diagnosis’ models were fitted using four chains with a total of 1250 iterations and warm-up of 500 iterations for each, giving 3000 samples from the posterior distribution of the parameter set, and convergence of the chains to a stable posterior distribution was checked. The four sample chains were run in parallel, and the required ‘wall-clock’ time for the most complex model was 18 hours and 50 minutes.

Further analysis of the posterior distributions of the true date of HIV infection in each patient was carried out for the model with varying incidence before and during the analysis window, with average diagnosis delay divided by ethnicity subgroup. This showed that a large majority of infections occurred either during or within 5 years prior to the analysis window (Figure D1), supporting the use of a model for HIV incidence that could plausibly cover this period.

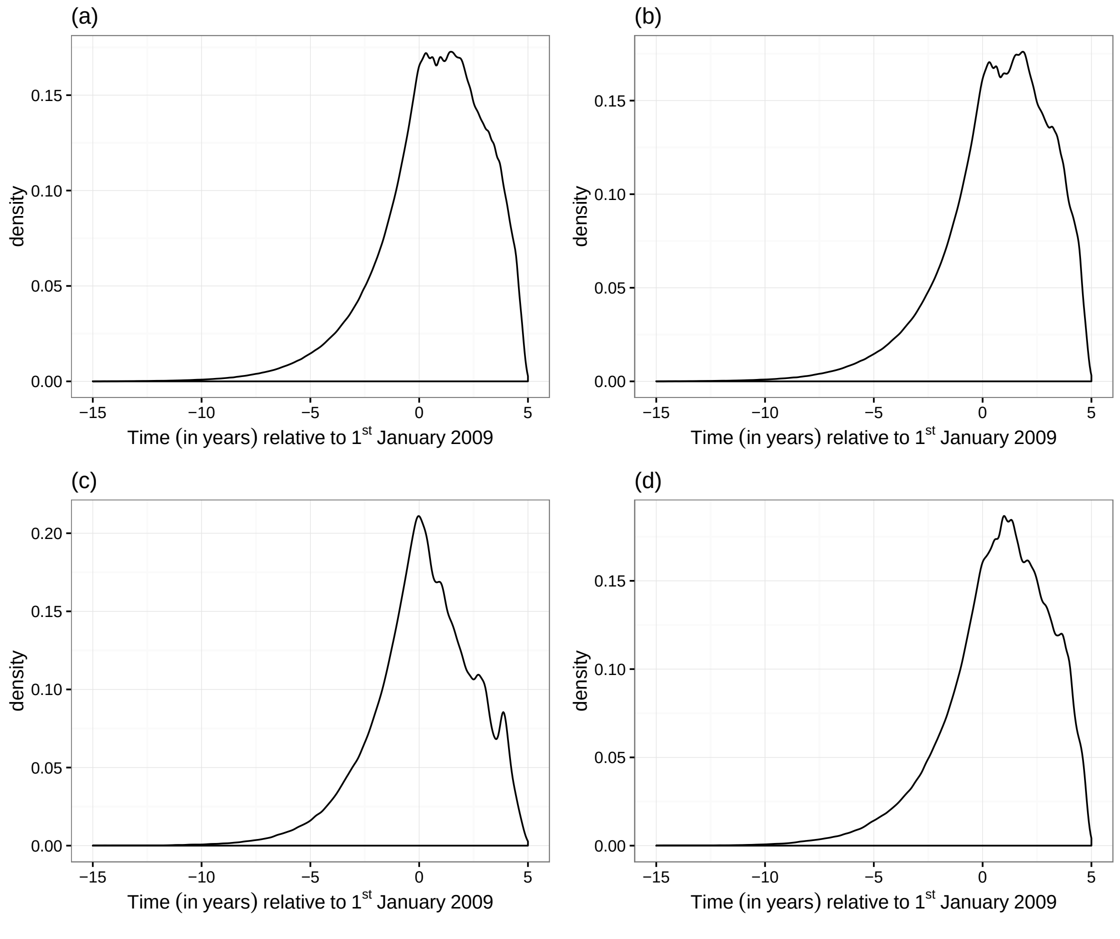

Figure D1: Plots of the pooled posterior density of infection times of patients included in the analysis. Plots are shown for the model with varying incidence during the window period and differences in delay-to-diagnosis distribution between groups, for all patients pooled (a) and for patients of white (b), black (c) or other (d) ethnic classification.

D.1 Predictions in individual seroprevalent patients

To demonstrate the use of the modelling framework in individual patients we explore predictions regarding the timing of HIV infection in two illustrative examples from the seroprevalent cohort. The first of these patients was randomly selected and the second was chosen because their clinical data provides strong evidence of recent infection.

Patient 1 was diagnosed with HIV in 2013 and a viral sequence sampled at time of diagnosis showed ambiguous nucleotide calls at 0.66 % of positions. CD4 cell counts of 300 and 295 cells/μL and $\log_{10}$ VL of 4.5 and 4.8 copies/mL were recorded at time of diagnosis and 40 days later, respectively. The genetic and biomarker data do not provide strong evidence regarding the exact timing of infection in this case, and when a uniform prior is used the posterior distribution obtained indicates that infection could plausibly have occurred at any point in the patient's adult life (Figure D2b). However, when information regarding the delay-to-diagnosis distribution is pooled across subgroups of patients the posterior distribution obtained indicates that the infection is very likely to have been acquired within the 5 years prior to the date of diagnosis (Figure D2a).

Patient 2 was diagnosed with HIV in 2009 and a viral sequence sampled 12 days after diagnosis showed no ambiguous nucleotide calls. The first three CD4 cell counts obtained were 615, 875 and 800 cells/μL at 12, 140 and 260 days after diagnosis, and the first three $\log_{10}$ VL measurements were 5.5, 2.8 and 3.4 copies/mL at 12, 140 and 430 days after diagnosis. The combination of no sequence ambiguity, high CD4 count and steep decrease in VL all indicate that diagnosis was close to the date of infection, and this is reflected in the posterior distribution for the timing of infection whether or not the delay-to-diagnosis distribution was explicitly modelled (Figure D2c and d).

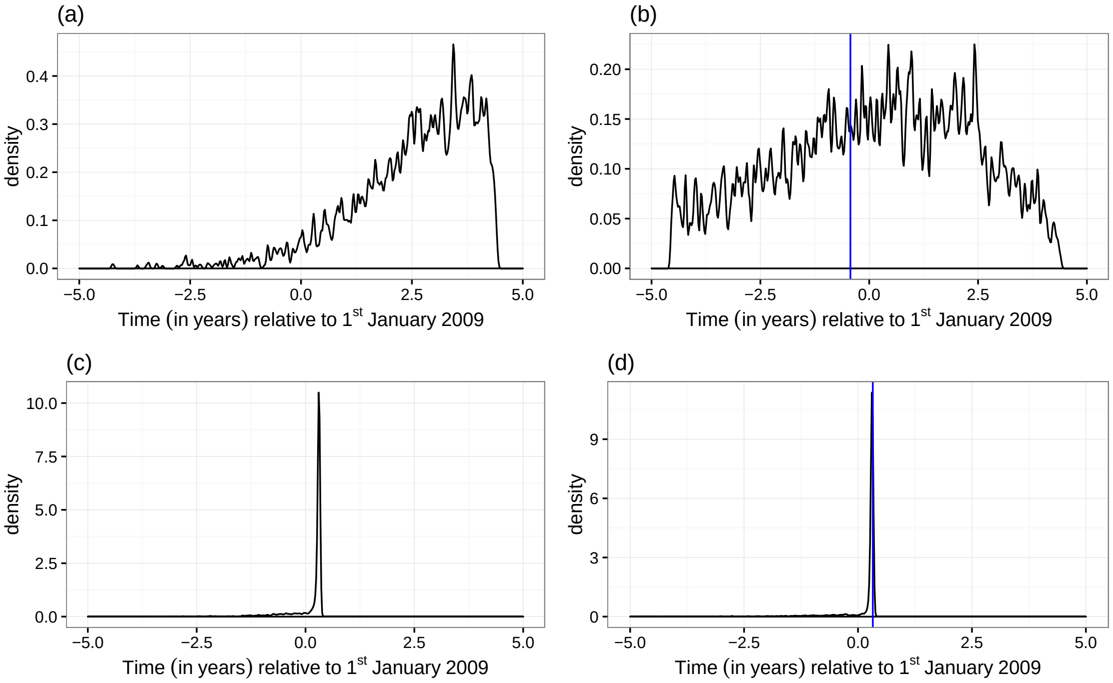

Figure D2: Illustrative plots of the posterior distributions of date of HIV infection in Patient 1 ((a) and (b)) and Patient 2 ((c) and (d)) given their sequence ambiguity, CD4 and viral load (VL) data. (a) and (c) show the posterior distribution from the full model with ethnic group-specific exponential distribution for delay to diagnosis and allowing changes in HIV incidence prior to and during the analysis window. (b) and (d) show the posterior distribution obtained from a patient-by-patient analysis with uniform prior distribution for the timing of HIV infection between the patient's 16th birthday and the date of diagnosis; in these plots the point estimate of date of infection obtained by CD4 back-estimation is also shown (vertical blue line). Kernal density smoothing of samples from the posterior distribution has been employed, using a Gaussian kernal with bandwidth of 1 week; a smoother estimate of the posterior distribution could be obtained by generating a greater number of samples from the posterior distribution in each case.

E Repeated simulation analysis without truncation of observation times

For both CD4 back-estimation and the use of our full biomarker model with uniform priors for the diagnosis delay, predictions are generated independently in each simulated patient. However for the methodology developed that includes the full biomarker model and an exponential survival model for delay to diagnosis times, predictions of the diagnosis delay in individual patients are affected by the distribution fitted to the cohort as a whole. To evaluate the performance of this methodology across multiple cohorts, we generated an additional 100 cohorts of 2000 simulated patients and refitted the model and evaluated individual-level predictions for each. The results obtained were consistent with those for the single simulated cohort presented in the main text of the paper (Table E1).

Table E1: Summary of 100 repetitions of the simulation analysis of 2000 patients without truncation of observation times. Our methodology was applied using an exponential survival model for diagnosis delays.

	n/N or mean±SD*
Successful model fit	97/100
Exponential dist. rate para.	
Posterior expectation	0.509 ± 0.019
Coverage of 95 % CrI	95/97
Mean absolute error (years)	1.047 ± 0.025
Bias (mean error) (years)	-0.0017 ± 0.056

*Mean and SD are given treating the result summary for each simulated cohort as a single observation.

References

[1] Pantazis N, Touloumi G, Walker AS, and Babiker AG. Bivariate modelling of longitudinal measurements of two human immunodeficiency type 1 disease progression markers in the presence of informative drop-outs. *Journal of the Royal Statistical Society: Series C (Applied Statistics)*, 54:405–423, 2005.

[2] Stirrup OT, Babiker AG, Carpenter JR, and Copas AJ. Fractional Brownian motion and multivariate-t models for longitudinal biomedical data, with application to CD4 counts in HIV-patients. *Statistics in Medicine*, 35:1514–1532, 2016.

[3] Stirrup OT, Babiker AG, and Copas AJ. Combined models for pre- and post-treatment longitudinal biomarker data: an application to CD4 counts in HIV-patients. *BMC Medical Research Methodology*, 16:121, 2016.

[4] Thiébaut R and Jacqmin-Gadda H. Mixed models for longitudinal left-censored repeated measures. *Computer Methods and Programs in Biomedicine*, 74:255–260, 2004.

[5] Thiébaut R, Jacqmin-Gadda H, Babiker A, Commenges D, and CASCADE Collaboration. Joint modelling of bivariate longitudinal data with informative dropout and left-censoring, with application to the evolution of CD4+ cell count and HIV RNA viral load in response to treatment of HIV infection. *Statistics in Medicine*, 24:65–82, 2005.

[6] Tobin J. Estimation of relationships for limited dependent variables. *Econometrica*, 26:24–36, 1958.

[7] Stan Development Team. Stan Modeling Language User's Guide and Reference Manual; Version 2.14.0. `http://mc-stan.org/documentation/`, December, 2016. [Online; accessed 30 May 2017].

[8] Lewandowski D, Kurowicka D, and Joe H. Generating random correlation matrices based on vines and extended onion method. *Journal of Multivariate Analysis*, 100:1989–2001, 2009.

[9] OT Stirrup, AJ Copas, AN Phillips, MJ Gill, RB Geskus, G Touloumi, J Young, HC Bucher, and AG Babiker. Predictors of CD4 cell recovery following initiation of antiretroviral therapy among HIV-1 positive patients with well-estimated dates of seroconversion. *HIV Medicine*, 19:184–194, 2018.

[10] Carpenter B, Lee D, Brubaker MA, Riddell A, Gelman A, Goodrich B, Guo J, Hoffman M, Betancourt M, and Li P. Stan: A Probabilistic Programming Language. *Journal of Statistical Software*, 76, 2017.

[11] Gelman A. A continuous hinge function for statistical modeling. `http://andrewgelman.com/2017/05/19/continuous-hinge-function-bayesian-modeling/`. [Online; accessed 30 May 2017].

[12] Gelman A and Rubin DB. Inference from iterative simulation using multiple sequences. *Statistical Science*, 7:457–472, 1992.
